# Tempol improves neuroinflammation and delays motor dysfunction in a mouse model (SOD1^G93A^) of ALS

**DOI:** 10.1186/s12974-019-1598-x

**Published:** 2019-11-14

**Authors:** Gabriela Bortolança Chiarotto, Luciana Politti Cartarozzi, Matheus Perez, Natalia Perussi Biscola, Aline Barroso Spejo, Fernanda Gubert, Marcondes França Junior, Rosália Mendez-Otero, Alexandre Leite Rodrigues de Oliveira

**Affiliations:** 1 Department of Structural and Functional Biology, Institute of Biology—Unicamp, Campinas, 13083-865 Brazil; 2 Department of Neurology, David Geffen School of Medicine at UCLA, Los Angeles, CA USA; 3 Instituto de Biofísica Carlos Chagas Filho, Centro de Ciências da Saúde, Sala G2-028, Universidade Federal do Rio de Janeiro, Cidade Universitária, Rio de Janeiro, RJ 21941-902 Brazil; 4 Department of Neurology, Faculty of Medical Sciences—Unicamp, Campinas, 13083-887 Brazil; 5 Laboratory of Nerve Regeneration University of Campinas—UNICAMP Cidade Universitária “Zeferino Vaz”, Rua Monteiro Lobato 255, Campinas, SP 13083970 Brazil

**Keywords:** Amyotrophic lateral sclerosis, Neuroinflammation, Tempol, ALS therapy

## Abstract

**Background:**

The development of new therapeutic strategies to treat amyotrophic lateral sclerosis (ALS) is of utmost importance. The use of cyclic nitroxides such as tempol may provide neuroprotection and improve lifespan. We investigated whether tempol (50 mg/kg) presents therapeutic potential in SOD1^G93A^ transgenic mice.

**Methods:**

Tempol treatment began at the asymptomatic phase of the disease (10th week) and was administered every other day until week 14, after which it was administered twice a week until the final stage of the disease. The animals were sacrificed at week 14 (initial stage of symptoms—ISS) and at the end stage (ES) of the disease. The lumbar spinal cord of the animals was dissected and processed for use in the following techniques: Nissl staining to evaluate neuronal survival; immunohistochemistry to evaluate astrogliosis and microgliosis (ISS and ES); qRT-PCR to evaluate the expression of neurotrophic factors and pro-inflammatory cytokines (ISS); and transmission electron microscopy to evaluate the alpha-motoneurons (ES). Behavioral analyses considering the survival of animals, bodyweight loss, and Rotarod motor performance test started on week 10 and were performed every 3 days until the end-stage of the disease.

**Results:**

The results revealed that treatment with tempol promoted greater neuronal survival (23%) at ISS compared to untreated animals, which was maintained until ES. The intense reactivity of astrocytes and microglia observed in vehicle animals was reduced in the lumbar spinal cords of the animals treated with tempol. In addition, the groups treated with tempol showed reduced expression of proinflammatory cytokines (*IL1β* and *TNFα*) and a three-fold decrease in the expression of *TGFβ1* at ISS compared with the group treated with vehicle.

**Conclusions:**

Altogether, our results indicate that treatment with tempol has beneficial effects, delaying the onset of the disease by enhancing neuronal survival and decreasing glial cell reactivity during ALS progression in SOD1^G93A^ mice.

## Background

Amyotrophic lateral sclerosis, with a global prevalence of 4–8 cases per 100,000 individuals, is the fourth leading cause of death by neurodegenerative diseases and follows Parkinson’s, Alzheimer’s, and Huntington’s disease. The ALS is characterized by selective and progressive degeneration of both upper and lower motoneurons [1–3]. The degenerative process is followed by intense muscular atrophy and sequential paralysis and death, usually by respiratory failure. The clinical signs emerge when neuronal degeneration reaches a critical point beyond compensatory mechanisms, generating denervation and muscular weakness. Initially, such partial degeneration is compensated for by the surviving neurons, which, through axonal sprouting, increase the size of the motor units. However, this mechanism eventually fails, and the cell bodies of the motoneurons become visibly abnormal and completely degenerate [4]. The disease progression is fast, with 50% of patients dying due to respiratory complications within 2 to 5 years after the onset of symptoms [5].

The discovery of SOD1 mutations led to the development of transgenic mouse models and provided a mechanism to investigate the disease pathogeneses. These animals present several aspects of the clinical profile observed in human patients with the disease, representing an excellent model for the study of pathological mechanisms of ALS [6].. Although the specific mechanism responsible for inducing ALS has not yet been elucidated, the pathophysiology of the disease appears to be multifactorial, including glutamate excitotoxicity, oxidative stress, mitochondrial dysfunction, altered protein control, and neuroinflammation, which are pivotal features in both ALS in humans and in ALS mouse models [7–9]. Of note is the active role of non-neuronal cells, such as astrocytes, microglial, and T lymphocytes, which characterize ALS as a non-cell autonomous disease [10]. In physiological conditions, astrocytes and microglia play important functions in the maintenance and protection of the central nervous system (CNS). They participate in several mechanisms, such as providing trophic factors, regulating glutamate concentration and controlling synaptic function [11]. Importantly, it has been shown that astrocytes and microglia are capable of modifying the disease progression of SOD1 transgenic mice [12, 13]. Despite evidence regarding several disease mechanisms, the ALS etiology remains unknown.

Although ALS has been described for more than a century, until last year, riluzole was the only drug approved by the FDA for ALS treatment. More recently, the FDA approved an antioxidant edaravone for the treatment of the disease, and both drugs present a modest increase in the survival of patients. Therefore, the discovery of new therapeutic strategies and drugs that can act in different pathways to promote a better quality of life for patients is urgently needed. The class of cyclic nitroxides has become a possible candidate for the treatment of neurologic disorders in the central and peripheral nervous system. These drugs are multifunctional antioxidants and present low toxicity in vitro and in vivo [14–16]. Tempol (4-hydroxy-TEMPO) is considered a cyclic nitroxide with low molecular weight and excellent cellular permeability. Although tempol is mainly characterized as an antioxidant, several studies demonstrated other effects in different pathological conditions of the nervous system, including anti-apoptotic, anti-inflammatory, immunomodulatory, and therapeutic proprieties [17–23]. It is also established that tempol restores muscular force in normal and dystrophic animals [24, 25], demonstrating that tempol can be considered a candidate for the treatment of neurodegenerative diseases. In this way, the aim of the present study was to investigate the neuroprotective effects of tempol for the treatment of ALS in a transgenic mouse model.

## Methods

### Ethics statements

All animal procedures were conducted according to the guidelines of the Brazilian College for Animal Experimentation. Experiments and animal handling were approved by the Institutional Committee for Ethics in Animal Experimentation (Committee for Ethics in Animal Use, Institute of Biology, CEUA/IB/UNICAMP), protocols n° 3304–1 and 4501–1/2017.

### Mice

**Fig. 1:**
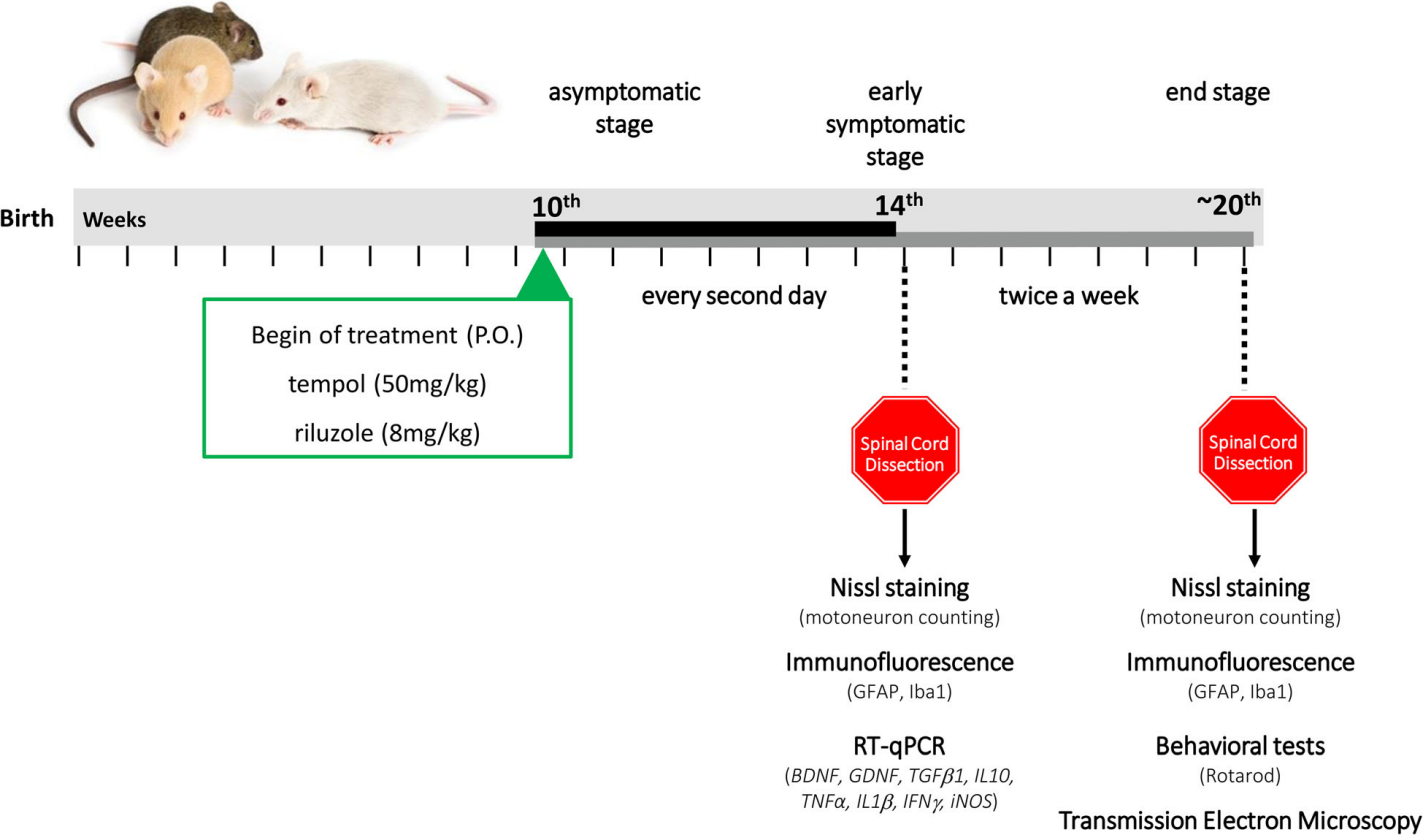
Experimental design, treatments, and methods employed to test the efficacy of tempol on the early symptomatic stage of ALS in the transgenic SOD1^G93A^ mouse model

The strain used was B6SJLTg (SOD1^G93A^), which was developed by Gurney et al. 1994, and it carries a mutant allele of human SOD1 containing the Gly 93 ➔ Ala substitution. The breeding pairs were originally obtained from the ALS Foundation through Dr. R. Brown (University of Massachusetts) and subsequently donated by Dra. Rosália Mendez-Otero (Instituto de Biofísica Carlos Chagas Filho, UFRJ). The colony was maintained by crossing transgenic males with wild-type female mice. The genotyping of animals and the number of human SOD1 transgenic copies was assessed as described in the Jackson Laboratory manual (Tables 1 and 2). These animals are characterized by presenting disease onset at approximately 90 days and survival at 130 days (Fig. 1). For this study, female and male mice were used in equivalent numbers.

**Table 1:** Primer sequences used for the genotyping of all animals

Primers	Sequence
Human SOD forward	5′ CAT CAG CCC TAA TCC ATC TGA 3′
Human SOD reverse	3′ TCT TAG AAA CCG CGA CTA ACA ATC 5′
Mouse SOD forward	5′ GCA ATC CCA ATC ACT CCA CAG 3′
Mouse SOD reverse	3′ GTC CAT GAG AAA CAA GAT GAC 5′

**Table 2:** Primer sequences used to assess the copy number of human SOD1 in transgenic mice

Primers	Sequence
IMR1544	CAC GTG GGC TCC AGC ATT
IMR3580	TCA CCA GTC ATT TCT GCC TTT G
IMR9655	GGG AAG CTG TTG TCC CAA G
IMR9666	CAA GGG GAG GTA AAA GAG AGC

### Drugs and treatments

The treatment with 50 mg/kg tempol (Sigma-Aldrich 176,141) or 8 mg/kg riluzole (Sun Pharmaceutical Ind. Ltd., India) started on the 10th week of life and was performed orally (gavage). The animals of the vehicle group received water alone instead of drug treatment. Between the 10th and 14th weeks, the animals were treated every other day. After this time point, the treatment continued twice a week until the end-stage of the disease. The doses employed in this study were based on previous studies [23, 26].

### Disease progression assessments

The progression of the disease was evaluated in all transgenic mice twice a week from the 10th week of age until end-stage by a researcher blinded to the treatment. The parameters measured included body weight, latency to fall in the Rotarod test, and neurological score.

#### Survival of transgenic mice

The survival of the animals was considered to be the age at which the animals were unable to return to the quadruple position within 30 s after being placed in dorsal decubitus.

#### Evolution of disease

Bodyweight measurements were used to determine disease onset and the symptomatic stage. All mice were weighed twice a week beginning on the 10th week until the endpoint. Disease onset was established as the maximum weight recorded for each animal retrospectively. The symptomatic stage was considered when the animals presented a loss of 10% of their maximum weight [27]. The difference between the age of onset and the age of euthanasia was used as a measurement of disease progression. The neurological score of 0 to 4 was evaluated as described in the strain manual of *Jackson Laboratory:* Working with ALS Mice *Guidelines for preclinical testing & colony management* from the Jackson Laboratory (http://jackson.jax.org/rs/444-BUH-304/images/Working_with_ALS_Mice.pdf).

#### Motor performance

Rotarod (EFF 412, Insight, Brazil) tests were conducted twice a week from the 10th week of age until the end stage, while possible. The mice had up to 8 min to remain in the rotating bar at a constant speed of 5 rpm. The time until the mice dropped from the cylinder was recorded.

### Tissue preparation for histological examination

Following the predetermined periods of treatment, namely, 14 weeks (onset of symptoms) and the end-stage of the disease (Table 3), the animals were anesthetized with Kensol (xylazine, König, Argentina, 10 mg/kg) and Vetaset (ketamine, Fort Dodge, USA, 50 mg/kg) and were subjected to transcardial perfusion with 0.1 M phosphate buffer saline (PBS), followed by fixative (paraformaldehyde 4% in 0.1 M phosphate buffer—PB; pH 7,4). The lumbar spinal cords were removed, postfixed in the same fixative solution for 12 h at 4 °C, washed with phosphate buffer (PB), and sequentially cryopreserved in 10%, 20%, and 30% PB-sucrose (12 h in each concentration). The samples were individually frozen in n-hexane, which was cooled in liquid nitrogen at − 35 °C. Transverse sections (12 μm thick) of lumbar spinal cords were obtained with a cryostat and transferred to gelatin-coated slides, dried at room temperature for 30 min, and stored at − 20 °C until utilization. After reaching room temperature, the sections were then stained with cresyl violet to count the motoneurons and subjected to immunolabeling.

**Table 3:** Experimental groups and distribution of animal numbers for each technique

Groups	n° immunohistochemistry and Nissl (14th)	n° immunohistochemistry and Nissl (end-stage)	n° RT-qPCR (14th)	n° TEM (end-stage)
NTG (non-transgenic)	6	6	5	3
Vehicle	6	6	5	3
Riluzole	6	6	5	3
Tempol	6	6	5	3

#### Nissl staining and motoneuron counting

The motoneurons were localized based on morphology and ventral horn location. The total number of motoneurons in the ventral horn of the lumbar spinal cord was counted in alternate sections of each specimen in approximately 20 sections. The interval between sections was 48 μm. Only cells with a visible nucleus and nucleolus were counted. To correct for the double-counting of motoneurons, because the same cell may be present in two sections, we used the Abercrombie’s formula [28] as follows:

$$ N=\mathrm{nt}/\left(t+d\right) $$

***N*** is the corrected number of counted neurons, *n* is the counted number of cells, *t* is the thickness of the sections (12 μm), and *d* is the average diameter of the cells. Due to the possibility of differences in cell size among experimental conditions, the value of *d* was calculated specifically for each experimental group. Thus, the diameter of 30 randomly chosen neurons present at the ventral horn lamina IX of each group was measured and the mean diameter obtained was applied to the formula (approximately 40 μm).

#### Immunofluorescence

Immunofluorescence was evaluated in three representative alternate sections of the lumbar spinal cord (12 μm thick). After blocking with 150 μL 3% BSA (bovine serum albumin) in 0.1 M PB for 45 min, the slides were incubated with rabbit anti-GFAP (Abcam 1:1500-AB7779) and rabbit anti-Iba1 (Wako, 1:700–01919741), diluted in an incubation solution containing 1.5% BSA and 0.2% Tween in 0.1 M PB and incubated for 4 h at room temperature. After washing with 0.01 M PB, the secondary antibodies (CY-3, anti-mouse, or anti-rabbit, Jackson Immunoresearch; 1:250) were applied and incubated for 45 min. The sections were then rinsed in 0.01 M PB and mounted in a mixture of glycerol/PB (3:1).

For quantification measurements, 3 representative images of the ventral horn of the lumbar spinal cord were captured from each animal for all experimental groups using a Leica fluorescence microscope (DM 5500, Wetzlar, Germany) equipped with a coupled digital camera (DFC 345 FX, Wetzlar, Germany) using the specific filters according to the secondary antibodies. A quantitative evaluation of labeling was carried out using the integrated density of pixel measurements in a fixed area corresponding to the ventral horn, as described by [29]. Quantification was performed with ImageJ software (version 1.33u, National Institutes of Health, USA). The integrated pixel density was calculated for each section, and the mean values for each experimental animal were computed. The data are presented as the mean ± standard error of the mean (SEM) for each group.

### Transmission electron microscopy

For ultrastructural analysis, the animals were killed at the end-stage of the disease with a lethal dose of halothane (Tanohalo, Cristália Chemicals, and Pharmaceuticals, Itapira-SP, Brazil), and the vascular system was transcardially perfused in a similar manner to that described in the “Tissue peparation for histological examination” section. After saline perfusion, the animals were fixed with a solution containing 2.5% glutaraldehyde and 0.5% paraformaldehyde in phosphate buffer 0.1 M (pH 7.4). The lumbar spinal cord was removed and stored overnight in the same fixative solution at 4 °C. The samples were trimmed and osmicated, dehydrated in ethanol and acetone, and embedded in Durcupan ACM (Fluka, Steinheim, Switzerland). The blocks were trimmed, and semithin sections (0.5 μm) were obtained and stained with 0.25% toluidine blue for light microscopy observation. Ultrathin sections (70 nm), from the right and left sides of the ventral horn, were made in an ultramicrotome (Leica Ultracut UCT Ultramicrotome), collected on formvar-coated single-slot grids, contrasted with uranyl acetate and lead citrate, and examined under a Tecnai G^2^ Spirit BioTwin (FEI, The Netherlands) transmission electron microscope operating at 80 kV.

#### Analysis of the ultrathin sections

Neurons with large cell bodies (> 35 μm in diameter) located in the ventral horn of the spinal cord were identified as α-motoneurons by the presence of *C*-type nerve terminals. The surface of these cells was then sequentially digitized at a magnification of 13,000× using a video camera (Eagle, FEI, Eindhoven, The Netherlands) connected to a computerized system. The images were then mounted together in vector graphics software (Adobe Photoshop CS4 Extended, Adobe Systems Incorporated, San Jose, CA, USA). A total of two motoneurons were evaluated per animal of each experimental group. Both the motoneurons and the neuropile were evaluated with respect to the degeneration process of the disease.

### RT-qPCR

The lumbar spinal cord was dissected and immediately frozen in liquid nitrogen and stored at − 80 °C. Total RNA was extracted using a specific RNeasy Lipid Tissue Mini Kit (Qiagen-cat n° 74,804), and reverse transcription was synthesized with 2.0 μg total RNA with the High Capacity cDNA Reverse Transcription Kit (Applied Biosystems–4,368,814) according to the manufacturer’s instructions*.* Following cDNA synthesis, real-time PCR was performed using a TaqMan Assay (Life Technologies) to evaluate the relative gene expression levels of neurotrophic factors (*BDNF* and *GDNF*), anti-inflammatory cytokines (*TGFβ1* and *IL10*) and pro-inflammatory cytokines (*TNFα*, I*L1β*, *IFNγ*, and *iNOS*) (Table 4). For the PCR template, cDNA specimens in triplicate were used with the TaqMan Gene Expression Master Mix (2×) (Life Technologies–PN 4369016) and TaqMan assays (primers + hydrolysis probes) for the genes listed in Table 4 in a volume of 20 μL, employing 45 cycles for amplification at 95 °C for 10 min, followed by 95 °C for 15 s and 60 °C for 1 min. The reference gene was carefully selected based on unchanged expression under several experimental conditions. The *HPRT1* reference gene was labeled with a VIC fluorophore and the target genes were labeled with a FAM fluorophore. The entire procedure for the quantitative PCR was performed on the instrumentation platform MX3005P (Agilent, Santa Clara, CA, USA), and the results were calculated with MxPro software (Agilent). For statistical analysis, the mean values of the three measurements for each animal were used as individual data for the relative quantification of the genes of interest using the 2^−ΔΔCt^ method [30].

**Table 4:** TaqMan assays used in the RT-qPCR analysis

Genes	Code
*HPRT1*	Mn01545399_m1
*IL1β*	Mn00434228_m1
*IL10*	Mn01288386_m1
*IFNγ*	Mn01168134_m1
*TGFβ1*	Mn01178820_m1
*TNFα*	Mn00619515_m1
*iNOS*	Mn00599849_m1
*BDNF*	Mn04230607_s1
*GDNF*	Mn00599849_m1

### Statistical analysis

For the survival analysis, Kaplan-Meier curves and log-rank tests (Mantel-Cox) were employed. For quantitative data analysis of neuronal survival, immunohistochemistry, and RT-qPCR, one-way ANOVA were used, followed by intergroup difference post hoc evaluation with Tukey’s test (parametric data). The Rotarod and body weight evaluations were analyzed using two-way ANOVA and the post hoc Bonferroni’s test. The level of statistical significance was 95% (*p* < 0.05).

## Results

### Tempol delays the bodyweight loss and the motor deficit in the Rotarod test associated with ALS progression

**Fig. 2:**
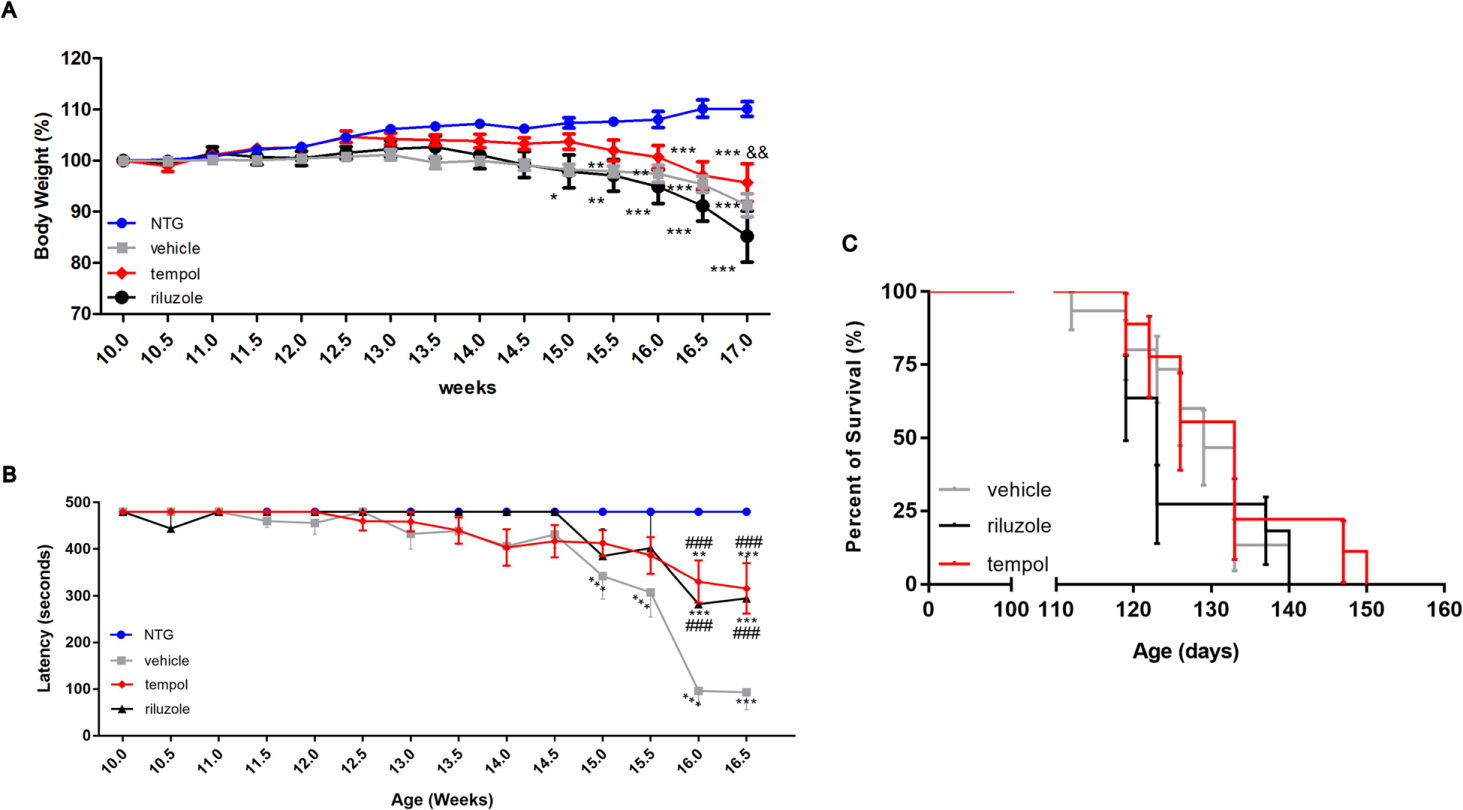
Behavioral analysis and survival. Tempol treatment delayed body weigt loss (**a**) and improved motor performance in the Rotarod test (**b**) compared to SOD1^G93A^ mice treated with vehicle or riluzole. Each point in the graphs represents the mean ± SEM. Data were analyzed by two-way ANOVA for repeated measures, followed by a post hoc Bonferroni test. Treatment with tempol showed no significant change in survival (**c**) compared to SOD1^G93A^ mice treated with vehicle. The results were evaluated with the log-rank test *p* = 0.5088. *** vs NTG, ### vs vehicle (*p* < 0.0001); && vs riluzole (*p* < 0.001)

The behavioral analyses (body weight and Rotarod test) were performed with experimenters blinded to treatment groups twice a week, starting at 10 weeks of age until 17 weeks (advanced symptomatic stage). The data obtained from the behavioral tests revealed an earlier loss of body weight (12 weeks) in the SOD1^G93A^ mice treated with riluzole and vehicle compared to the NTG group, starting from the asymptomatic phase of the disease (Fig. 2a). The group treated with tempol showed alterations 1 week later (13 weeks). This delayed weight loss was present in the tempol-treated group until the advanced stages of disease (17 weeks) and was significantly different from the riluzole-treated group (*p* < 0.001). In addition, the results obtained from the Rotarod test demonstrated improved motor performance in the animals treated with riluzole and tempol compared with that in the vehicle group (Fig. 2b). The behavioral improvement observed correlated positively on the survival of the animals, as shown in Fig. 2c. Thus, the group treated with tempol showed a trend towards increased lifespan compared with the other groups.

### Tempol attenuates neuronal loss during ALS progression in SOD1^G93A^ transgenic mice

Spinal MNs count was performed in the lumbar spinal cord, particularly in the segments related to the sciatic nerve. This region is characteristically afflicted by the disease in SOD1^G93A^ ALS mice. Thus, large lumbar MNs within the ventral horn lamina IX were counted by using Nissl staining at each time point. The percent change in the number of motoneurons in the transgenic SOD1^G93A^ mice and in the non-transgenic mice was calculated, which represents the ratio of neuronal survival during ALS progression.

**Fig. 3:**
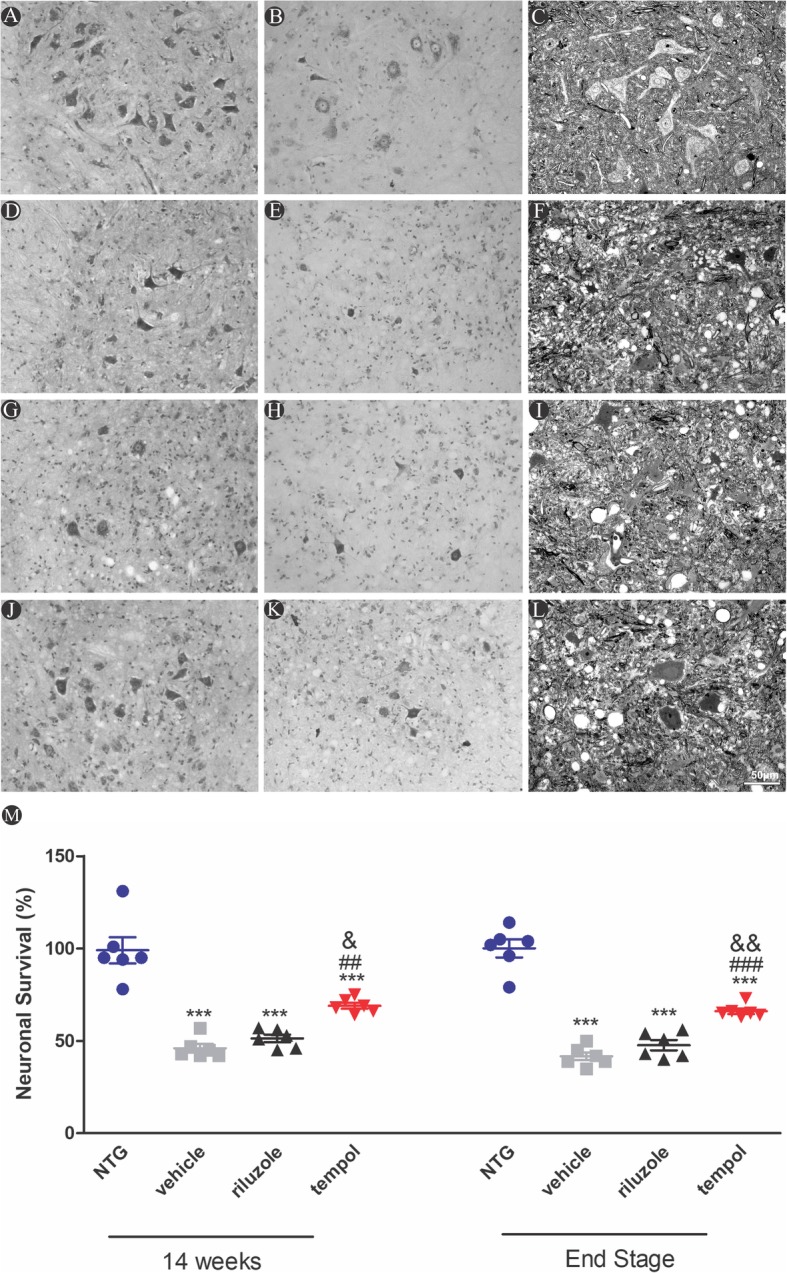
Histological sections of the lumbar spinal cord of non-transgenic and SOD1^G93A^ transgenic mice stained with cresyl violet (**a, b, d, e, g, h, j, k**) and semithin sections of the ventral horn of the spinal cord stained with toluidine blue (**c**, **f**, **i**, **l**). Scale bar: 50 μm. **m** Neuronal survival percentage, 14 weeks and end stage of ALS. Data were analyzed by one-way ANOVA, followed by post hoc Tukey’s test. ***p < 0.0001 vs NTG; ##*p* < 0.001 and ###*p* < 0.0001 vs vehicle; &&*p* < 0.001 and &&&*p* < 0.0001 vs riluzole

Despite all the drastic alterations that result from the ALS degeneration process observed with TEM (Additional file 1: Fig. S1), the tempol treatment showed promising results. At 14 weeks, SOD1^G93A^ mice showed a reduction of MNs of approximately 58% (vehicle), 53% (riluzole), and 36% (tempol) with respect to non-transgenic (NTG) littermates. The neuroprotection observed with tempol treatment was maintained until the end-stage of the disease, with 64% neuronal survival. These results were correlated with the improved ultrastructural preservation of the ventral horn of the spinal cord the tempol group compared to the ultrastructure of the vehicle and riluzole groups. The following percent changes in the number of motoneurons for each group were observed at 14 weeks: NTG (99% ± 7.1%, mean ± standard error); vehicle (46% ± 2.3%); riluzole (51% ± 2.0%); and tempol (69% ± 1.6%). The following percent changes for each group were observed at *end-stage*: NTG (100% ± 4.8%); vehicle (41.7% ± 2.1%); riluzole (46.8% ± 2.7%) and tempol (64.8% ± 0.5%) (Fig. 3). The total number of counted motoneurons is also provided (Additional file 2: Table S1). Importantly, within each group, male and female presented similar numbers of MNs (Additional file 3: Fig. S2).

### Tempol immunomodulates glial cell reactions during ALS progression

**Fig. 4:**
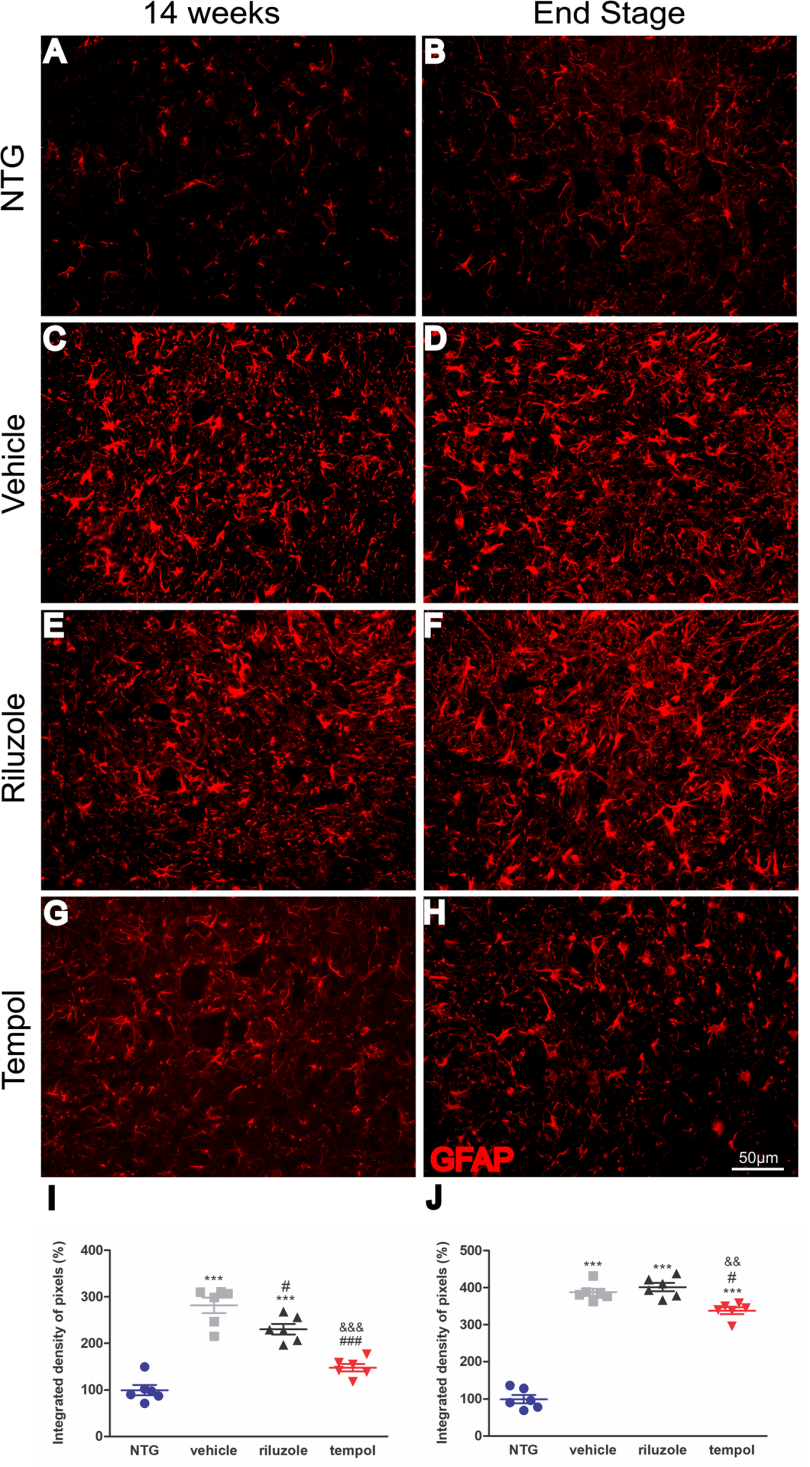
Images of astrocyte activation in the anterior horn of the spinal cord. The immunolabeling of GFAP was quantified in the early symptomatic phase (week 14) (**a**, **c**, **e**, **g**) and at the end stage (**b**, **d**, **f**, **h**). Scale bar: 50 μm. Graphical representation of percent GFAP expression at 14 weeks (**i**) and end-stage (**j**) of ALS. Data were analyzed by one-way ANOVA, followed by post hoc Bonferroni test. ***p < 0.0001 vs NTG; ###*p* < 0.0001 vs vehicle; &&*p* < 0.001 vs riluzole

It has been suggested that glial cells act as the protagonist of neuroinflammation during motor neurodegeneration in ALS. Therefore, we evaluated glial cell activation in the lumbar spinal cord during ALS progression (14 weeks and end-stage of disease). As expected, the immunohistochemical detection of GFAP showed strong astrogliosis in the spinal cord of SOD1^G93A^ mice compared with that of non-transgenic littermates (*p* < 0.0001). The analysis showed lower astrocyte activation (GFAP^+^) in tempol-treated mice than in vehicle-treated (134%) and riluzole-treated (83%) mice at the early symptomatic stage (14 weeks). The activation of astrocytes progressed until the end-stage of the disease. The following percent changes in GFAP quantification for each group were observed at 14 weeks: NTG (99.3% ± 10.8%, mean ± standard error); vehicle (281.5% ± 16.5%); riluzole (230.3% ± 11.3%); tempol (147.3% ± 8.2%); *end-stage*: NTG (99.5% ± 11.01%, mean ± standard error); vehicle (387.7% ± 9.5%); riluzole (401.5% ± 11.04%); tempol (337.8% ± 8.8%) (Fig. 4). Of note, within each group, male and female presented comparable astroglial reaction (Additional file 3: Fig. S2).

Similar data were obtained with respect to microglial activation (Iba1^+^). Tempol treatment decreased microglial reactivity by 83% in the ventral horn of the spinal cord at 14 weeks compared with the vehicle treatment. In the end-stage of ALS, the reduction in microgliosis was maintained by tempol (107%) in the ventral horn of the spinal cord. This effect was also observed in the intermediate region with a reduction of 104% compared with the vehicle group and 46% compared with the riluzole group. Additionally, in the dorsal region, 48% and 30% less reactivity was observed in the tempol group compared with the reactivity in the vehicle and riluzole groups, respectively.

**Fig. 5:**
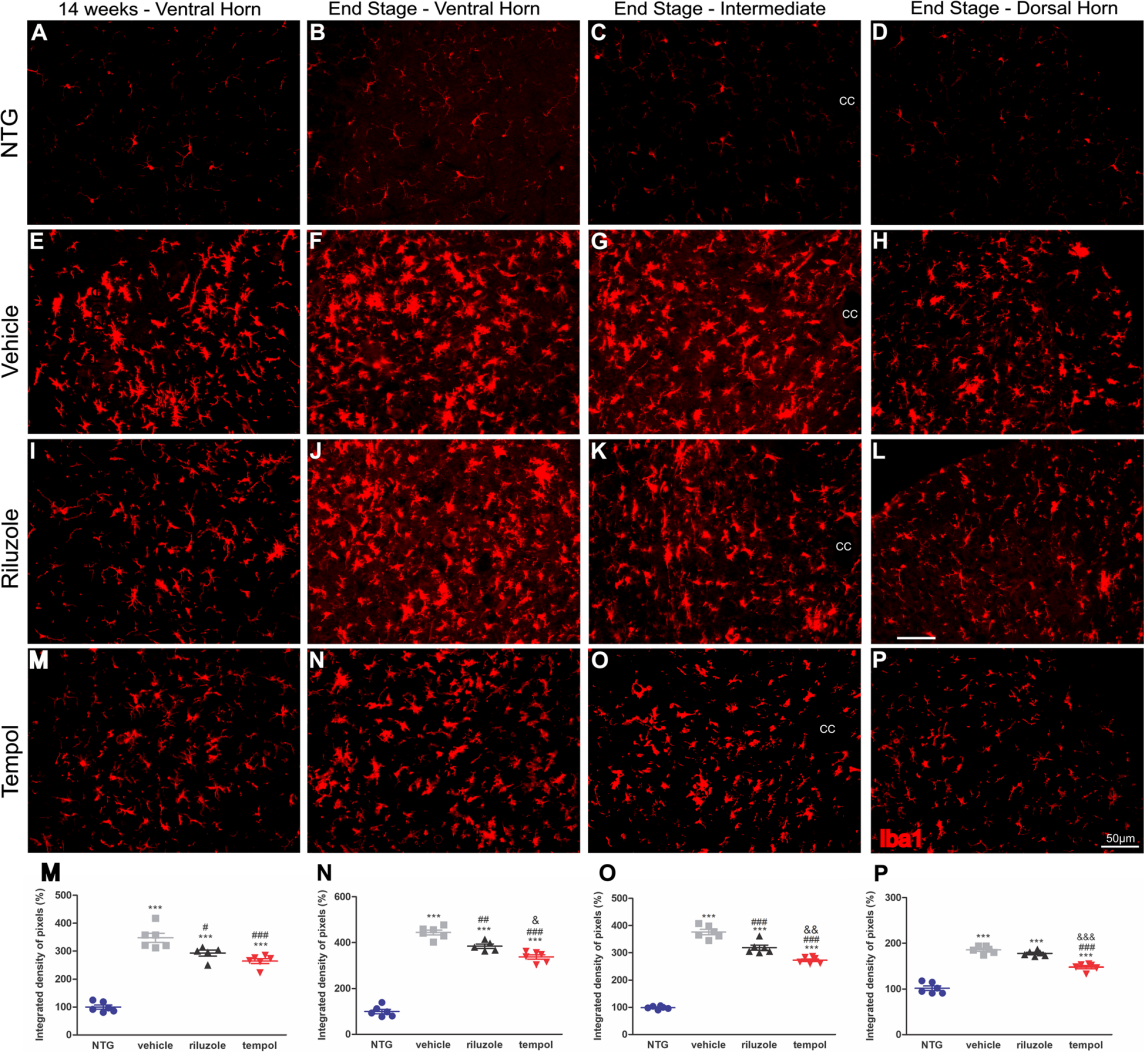
Images show microglial activation in the anterior horn of the spinal cord. The immunolabeling of Iba1 was quantified in the early symptomatic phase (week 14) and at the end stage (**a–p**). Scale bar: 50 μm. Graphical representation of percent Iba 1 expression at 14 weeks (**m**) and end-stage (**n,** ventral horn), (**o,** intermediate region), (**p**, dorsal horn) of ALS. Data were analyzed by one-way ANOVA, followed by post hoc Bonferroni test. ***p < 0.0001 vs NTG; #*p* < 0.05, ##*p* < 0.001, and ###*p* < 0.0001 vs vehicle; &*p* < 0.05, &&*p* < 0.001, and &&&*p* < 0.0001 vs riluzole

The following percent changes in Iba1 quantification for each group were observed at 14 weeks: NTG (100% ± 7.3%, mean ± standard error); vehicle (347.8% ± 16.2%); riluzole (292.8% ± 11.1%); and tempol (264.2% ± 13.68%). The following percent changes were observed at the *end-stage*: NTG (99.8% ± 9.3%), vehicle (444.4% ± 10.4%), riluzole (384.2% ± 9.5%), and tempol (337% ± 9.5%) in the ventral horn; NTG 99% ± 2.3%), vehicle (376.2% ± 9.7%), riluzole (318.7% ± 9.3%), and tempol (272.5% ± 4.8%) in the intermediate region; and NTG (102% ± 4.8%), vehicle (185.7% ± 3.2%), riluzole (177.7% ± 2.4%), and tempol 147.8% ± 3.2%) in the dorsal horn (Fig. 5). Importantly, within each group, male and female presented similar degree of microglial reactivity (Additional file 3: Fig. S2).

### Tempol decreases the activation of neurotrophic factors in the early symptomatic stage of ALS

**Fig. 6:**
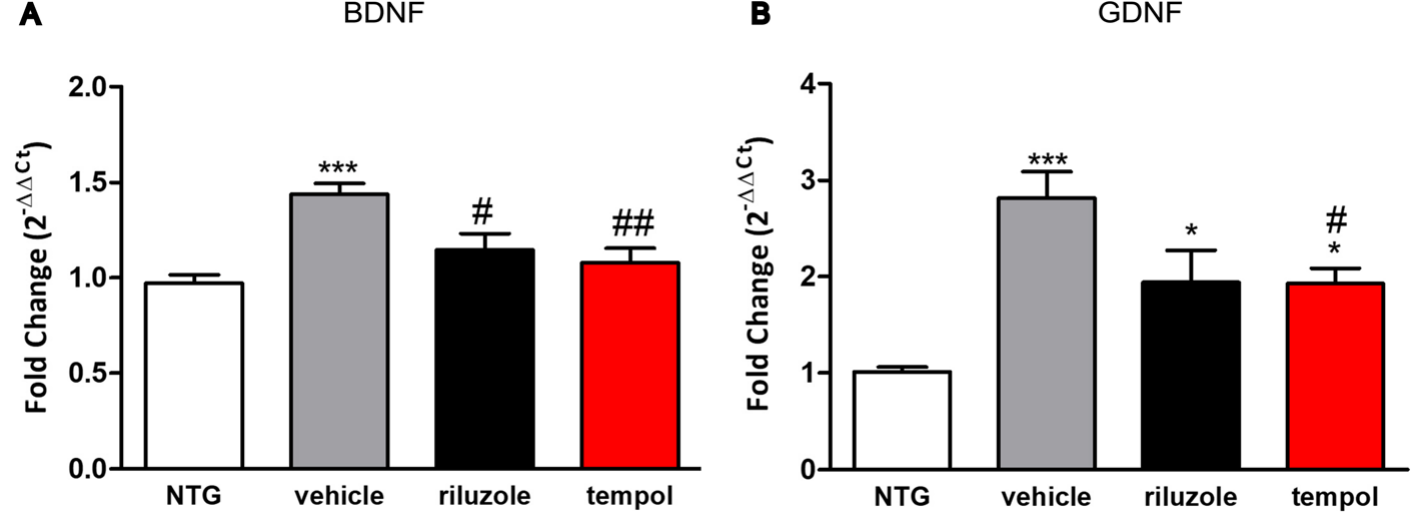
Relative expression of the neurotrophic factors *BDNF* (**a**) and *GDNF* (**b**). Data were analyzed by one-way ANOVA, followed by post hoc Tukey’s test. *p < 0.05 and ***p < 0.0001 vs NTG; #*p* < 0.05 and ##*p* < 0.001 vs vehicle

The gene expression of the neurotrophic factors *BDNF* and *GDNF* was assessed at 14 weeks by RT-qPCR. The *BDNF* gene expression increased in the vehicle group compared with the non-transgenic mice (*p* < 0.0001). However, riluzole and tempol treatments showed similar reduction in the *BDNF* expression when compared with vehicle group (*p* < 0.05 and *p* < 0.001). (NTG (0.97 ± 0.04), vehicle (1.4 ± 0.05), riluzole (1.1 ± 0.08), tempol (1.07 ± 0.07)) (Fig. 6a).

*GDNF* gene expression also increased in the transgenic mice groups compared with that of NTG; however, tempol treatment showed a reduction when compared with the vehicle group *p* < 0.05. NTG (1.01 ± 0.05), vehicle (2.81 ± 0.27), riluzole (1.94 ± 0.03), tempol (1.92 ± 0.16) (Fig. 6b).

### Tempol downregulates the gene expression of inflammatory cytokines in the early symptomatic stage of ALS

**Fig. 7:**
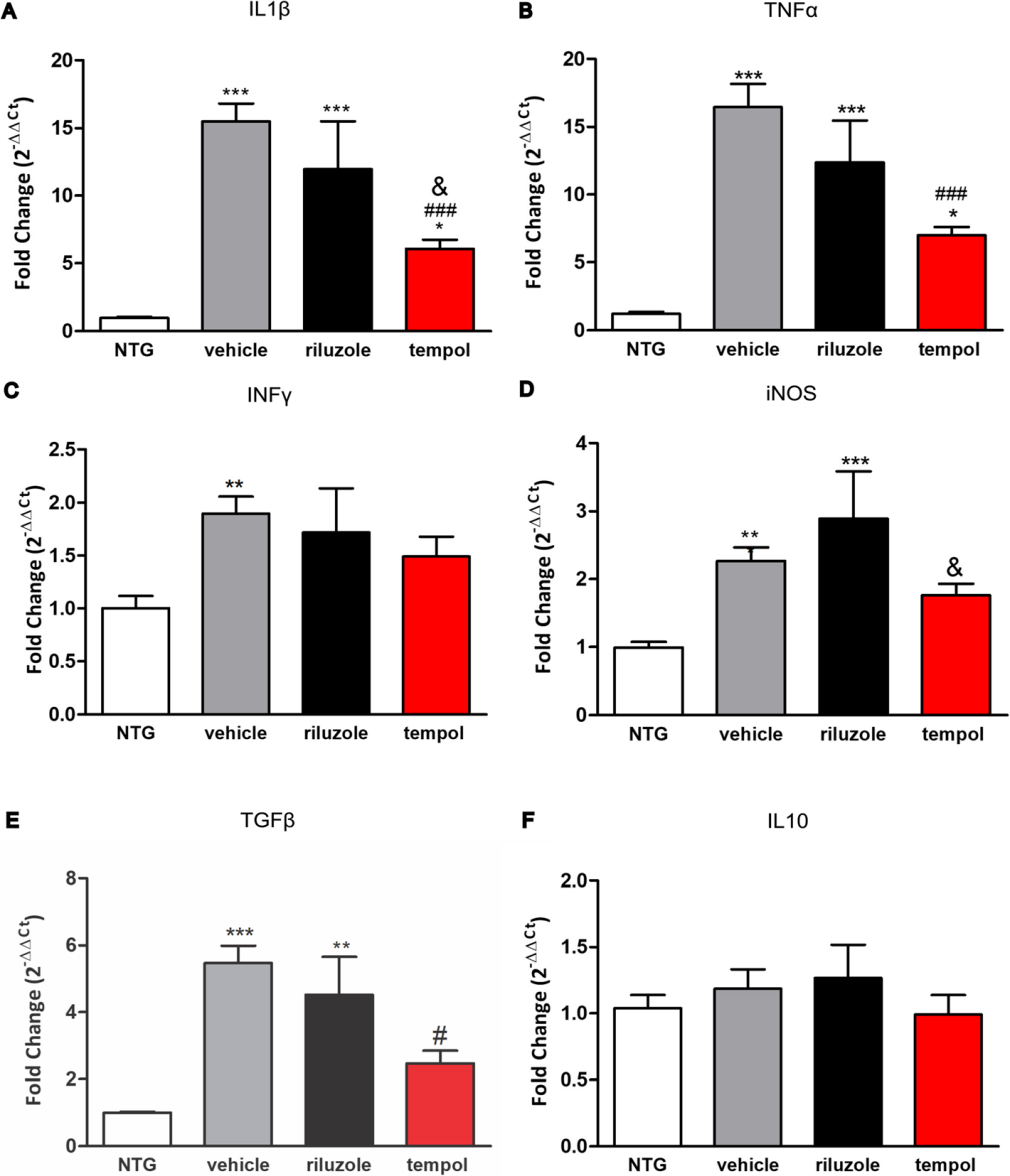
Relative expression of inflammatory cytokine s *IL1β* (**a**) and *TNFα* (**b**), *IFNγ* (**c**), *iNOS* (**d**), *TGFβ1* (**e**), and *IL10* (**f**). Data were analyzed by one-way ANOVA, followed by post hoc Tukey’s test. ***p < 0.0001, **p < 0.001, and *p < 0.05 vs NTG; ###*p* < 0.0001 and #*p* < 0.05 vs vehicle; &*p* < 0.05 vs. riluzole

In addition, the expression of pro- and anti-inflammatory cytokines was evaluated at 14 weeks. The *IL1β* transcript levels increased in transgenic mice: vehicle, riluzole, and tempol treatment by 14-, 10-, and 6-fold, respectively, compared to NTG mice. Tempol treatment promoted a 9-fold reduction compared with vehicle treatment *p* < 0.0001 and 6-*fold* reduction compared with riluzole treatment *p* < 0.05. NTG (0.96 ± 0.08), vehicle (15.5 ± 1.32), riluzole (11.97 ± 3.5), tempol (6.08 ± 0.65 (Fig. 7a).

Similar results were observed in *TNFα* gene expression, with higher levels in transgenic mice: vehicle, riluzole, and tempol treatment resulted in a 15-, 11-, and 6-fold increase, respectively, compared to NTG mice. Tempol again reduced the levels of *TNFα* by 9.5-*fold* when compared with vehicle (*p* < 0.0001; NTG (1.2 ± 0.14), vehicle (16.4 ± 1.7), riluzole (12.3 ± 3.1), and tempol (6.9 ± 0.61) (Fig. 7b).

The *IFNγ* transcript levels increase in vehicle group in respect NTG littermates (*p* < 0.001); however, no statistical differences were observed among the experimental groups NTG (1.0 ± 0.11), vehicle (1.8 ± 0.16), riluzole (1.71 ± 0.41), and tempol (1.49 ± 0.18) (Fig. 7c).

The gene expression of *iNOS* increased in the vehicle (*p* < 0.001) and riluzole (*p* < 0.001) groups compared to that of NTG mice. In contrast, the tempol group maintained *iNOS* levels close to the NTG group and reduced the *iNOS* levels in respect to riluzole group (*p* < 0.05). NTG (0.99 ± 0.08), vehicle (2.2 ± 0.20), riluzole (2.8 ± 0.69), and tempol (1.78 ± 0.17) (Fig. 7d).

*TGFβ1* gene expression increased in the vehicle (*p* < 0.0001) and riluzole (*p* < 0.001) groups compared to that of NTG mice. In addition, tempol treatment reduced the levels of *TGFβ1* by 3-fold with respect to vehicle group. NTG (1.0 ± 0.03), vehicle (5.4 ± 0.52), riluzole (4.5 ± 1.1), and tempol (2.4 ± 0.37), (Fig. 7e).

The *IL10* transcript levels were similar among all experimental groups (NTG (1.04 ± 0.09), vehicle (1.1 ± 0.14), riluzole (1.2 ± 0.24), tempol (0.99 ± 0.14)) (Fig. 7f).

## Discussion

Amyotrophic lateral sclerosis (ALS) is one of the most debilitating neurodegenerative diseases. Riluzole and edaravone are the only options to delay the course of the disease; however, they offer limited benefits to patients. Therefore, there is an urgent need to find new therapies to cure or at least delay the progression of the disease. The present study showed that oral administration of the antioxidant tempol, starting in the asymptomatic phase of ALS, delays motor dysfunction, and exerts immunomodulatory effects in SOD1^G93A^ mice, the most established mouse model for studying ALS. The improvement in behavioral tests was associated with neuroprotective effects in the spinal cord, which was correlated with increased motoneuron survival.

Neuroinflammation is the most evident pathological hallmark of ALS. Nevertheless, several studies suggest that this mechanism is not only a late consequence of motoneuron injury but may have a dual function, contributing to neuroprotection or leading to neurotoxicity. Initially, glial cells participate in the protective events aimed at supporting motoneuron survival. However, after disease onset, astrocytes and microglia acquire an aberrant phenotype, accelerating ALS progression and exacerbating neuronal degeneration. The threshold that determines the shift between protection and toxicity is unknown, although it is clear that the modulation of glial reactivity is a key strategy to avoid loss of motoneurons during the course of ALS. Our study showed the efficiency of tempol treatment to decrease astrogliosis and microglial reactions in the lumbar spinal cord. It is possible that the antioxidant actions of tempol reduced oxidative stress, positively influencing the inflammatory process during the installation of disease.

Our results showed the activation of glial reactivity beginning at the onset of ALS symptoms. At the same time point (14 weeks), the upregulation of the neurotrophic factors BDNF and GDNF indicated an effort to rescue diseased motoneurons. Tempol-treated transgenic mice displayed fewer neurotrophic factor gene transcripts, indicating reduced degenerative conditions in that group. Elevated levels of neurotrophic factors were observed in the lumbar spinal cord and limb muscles of ALS mice beginning at the asymptomatic stage and increasing progressively until the end stage of the disease [31, 32]. The same result was found in tissues of human ALS patients [33], suggesting the activation of a neuroprotective pathway through neurotrophic factor expression. Nonetheless, such a neuroprotective mechanism is not sufficient to avoid neuronal degeneration.

As expected, we found elevated levels of *TNFα* and *IL1β* pro-inflammatory cytokines produced by toxic microglia. These data are in accordance with the enhancement of glial reactivity in the initial symptomatic stage. Thus, astrocytes and microglia, under pathological conditions or in neurodegenerative diseases, release pro-inflammatory molecules, activating a signaling cascade that results in further damage of the CNS. Importantly, our present data suggest that tempol treatment after ALS onset can break the degenerative cycle of uncontrolled inflammation, inducing a predominantly protective astrocyte and microglia phenotype in the lumbar spinal cord of ALS mice.

Our results regarding *TGFβ1* expression were in line with the literature regarding ALS progression [34] demonstrated that the astrocytes of ALS patients and mice express *TGFβ1*, increasing the inflammatory response, which in turn accelerates disease progression. Additionally, the overproduction of *TGFβ1* results in decreased expression of insulin growth factor-I (*IGF-I*) by microglia and in a reduction of infiltrating T cells. Although we did not evaluate the expression of *TGFβ1* in astrocytes alone, our data suggest that the immunomodulatory role of tempol reduces astrogliosis as well as the levels of pro-inflammatory cytokines.

Altogether, our results emphasize that neuroinflammation has both beneficial and detrimental roles in neurodegenerative diseases. The challenge is to enhance the protective phase of neuroinflammation and to minimize its toxic effects. We have previously reported neuroprotective and anti-apoptotic effects of tempol after a neonatal lesion in the peripheral nervous system. Accordingly, other studies reported several positive effects of tempol in vitro and in vivo in different pathological conditions of the nervous system. The observed properties include inhibition of apoptotic events, anti-inflammatory effects, reduced glutamate release in pathologic conditions [18], modulation of *BDNF*, reduction of oxidative stress, and improvement of cognitive capacity in a model of Alzheimer’s disease [20]. In addition, therapeutic effects were reported in the EAE [19] and neuroprotection was reported in an in vitro model of Parkinson’s disease [21].

The present study shows that tempol is effective in reducing neuronal loss in ALS, even at the end-stage. Thus, we showed immunomodulatory effects on astrocytes and microglia, mostly in the initial symptomatic stage. Our results are in line with the gene expression analysis, which showed the downregulation of the pro-inflammatory cytokines *IL1β* and *TNFα*.

It is important to emphasize that the behavioral tests showed that tempol treatment delayed the motor deficit by 1 week compared to the vehicle-treated group. However, despite all the positive effects described, tempol treatment did not show a statistically significant effect on the overall survival of ALS mice. Contrasting results were reported by Linares et al. [35], who showed that treatment with tempol (26 mg/kg, i.p.), increased the lifespan of female SOD1^G93A^ mice. In our study, however, male and female mice were used in similar proportions to compose the different experimental groups. This may have obscured eventual gender-related effects [36]. However, in line with our findings, treatment with sodium valproate, another promising drug, although providing neuroprotection in SOD1^G86R^ mice did not extend lifespan. The authors suggested that such result was related with skeletal muscle denervation [37]. Thus, due to protocol, strain, and gender variations, conflicting results make challenging to determine the exact role of new drug candidates to treat ALS, difficulting the translation to the clinic. Nevertheless, the present data reinforce the therapeutic potential of multifactorial antioxidants, such as tempol, for the treatment of this particular motoneuron disease. Further studies are necessary to unveil the molecular basis of tempol, as well as eventual side effects of its chronic use.

## Conclusion

Overall, our study demonstrates that tempol can be considered a promising drug to treat ALS due to the improved immunomodulatory and anti-inflammatory effects compared to riluzole. Tempol showed neuroprotective, anti-inflammatory, and immunomodulatory properties throughout ALS progression, reducing body weight loss and improving motor performance in SOD1^G93A^ mice.

## Supplementary information

**Additional file 1: Figure S1.** Ultrastructural analysis of the ventral horn of the lumbar spinal cord at the end stage of ALS in transgenic SOD1^G93A^**.** (A) Cholinergic presynaptic terminal (Type C), necessary for identification of alpha motoneurons (18,500x). (B) Remaining spinal motoneuron (890x). (C) Atrophic motoneuron (890x). (D) Phagocytic microglia observed in the proximity of the neuronal body (2900x). (E) Protoplasmic astrocytes observed in the vicinity of the neuronal membrane. Projections of these cells were observed filling the space between the presynaptic terminals and the postsynaptic membrane (2900x). (F) Swollen mitochondria, showing the retraction of crests and rupture of internal membranes, which are characteristic of mitochondrial dysfunction (11.000x).

**Additional file 2: Table S1.** Number of motoneurons counted per spinal cord section (with Abercrombie’s correction, mean ± standard error, *n* = 6 per group).

**Additional file 3:** **Figure S2.** Evaluation of neuronal survival, astrocytosis and migroglial reaction in male and female mice from NTG, vehicle, riluzole-treated, and tempol-treated groups (14 weeks and end-stage). No gender differences were observed within each group/treatment (mean ± standard error).

## Data Availability

The datasets generated during and/or analyzed during the current study are available from the corresponding author on reasonable request.

## Abbreviations

ALS: Amyotrophic lateral sclerosis
BDNF: Brain-derived neurotrophic factor
CNS: Central nervous system
ES: End stage of the disease
FDA: Food and Drug Administration
GDNF: Glial cell-derived neurotrophic factor
GFAP: Glial fibrillary acidic protein
*IFNγ*: In*terferon gamma*
*IGF-1*: I*nsulin growth factor-I*
*IL1β*: *Interleukin 1 beta*
*iNOS*: *Inducible nitric oxide synthase*
ISS: Initial stage of symptoms
NTG: Non-transgenic
qRT-PCR: Quantitative real-time polymerase chain reaction
RPM: Rotation per minute
SOD: Superoxide dismutase
TEM: Transmission Electron Microscopy
*TGFβ1*: *Transforming growth factor beta 1*
*TNFα*: *Tumor necrosis factor alpha*
