# Supplementary figures and images for: Tempol improves neuroinflammation and delays motor dysfunction in a mouse model (SOD1G93A) of ALS
Source: J Neuroinflammation. 2019 Nov 14;16:218. doi: 10.1186/s12974-019-1598-x (PMC6857328; doi:10.1186/s12974-019-1598-x)

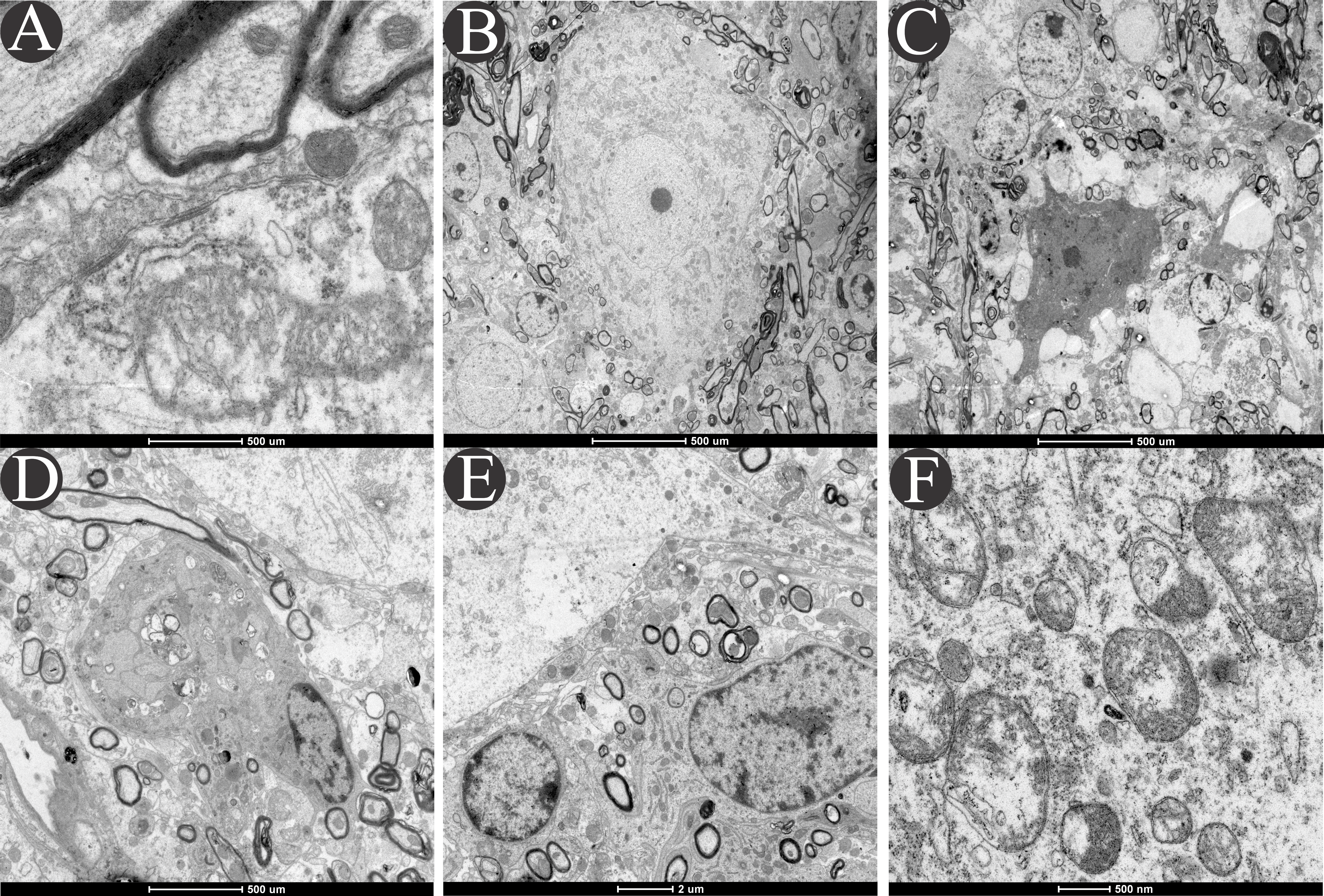

Supplement: Supplementary file 1 — Additional file 1: Figure S1. Ultrastructural analysis of the ventral horn of the lumbar spinal cord at the end stage of ALS in transgenic SOD1G93A. (A) Cholinergic presynaptic terminal (Type C), necessary for identification of alpha motoneurons (18,500x). (B) Remaining spinal motoneuron (890x). (C) Atrophic motoneuron (890x). (D) Phagocytic microglia observed in the proximity of the neuronal body (2900x). (E) Protoplasmic astrocytes observed in the vicinity of the neuronal membrane. Projections of these cells were observed filling the space between the presynaptic terminals and the postsynaptic membrane (2900x). (F) Swollen mitochondria, showing the retraction of crests and rupture of internal membranes, which are characteristic of mitochondrial dysfunction (11.000x). [file 12974_2019_1598_MOESM1_ESM.tif]
