# Supplementary material for: Tempol improves neuroinflammation and delays motor dysfunction in a mouse model (SOD1G93A) of ALS
Source: J Neuroinflammation. 2019 Nov 14;16:218. doi: 10.1186/s12974-019-1598-x (PMC6857328; doi:10.1186/s12974-019-1598-x)
Supplement: Supplementary file 2 — Additional file 2: Table S1. Number of motoneurons counted per spinal cord section (with Abercrombie’s correction, mean ± standard error, n = 6 per group). [file 12974_2019_1598_MOESM2_ESM.docx]

| Time | NTG | Vehicle | Riluzole | Tempol |
| --- | --- | --- | --- | --- |
| 14 weeks | 8.01±0.67 | 3.58±0.19 | 4.02±0.17 | 5.50±0.13 |
| End stage | 6.92±0.57 | 2.87±0.22 | 3.33±0.32 | 4.55±0.41 |
